# The prevalence of risk factors and pattern of obstructive coronary artery disease in young Indians (< 45 years) undergoing percutaneous coronary intervention: A gender-based multi-center study

**DOI:** 10.1016/j.ihj.2022.07.001

**Published:** 2022-07-16

**Authors:** Pankaj Jariwala, Alwala Padmavathi, Rahul Patil, Kamaldeep Chawla, Kartik Jadhav

**Affiliations:** a Yashoda Hospitals, Somajiguda, Hyderabad, Telangana 500082, India; b Gandhi Medical College, India; c Ruby Hall Clinic, Sangamvadi, Pune, Maharashtra 411001, India; d Sterling Hospitals, Race Course Road, Opposite Inox Cinema Hari Nagar, Circle West, Vadodara, Gujarat 390007, India

**Keywords:** Coronary artery disease, Percutaneous coronary intervention, Risk factors, Women

## Abstract

**Objectives:**

In a retrospective study, we aimed to explore the prevalence of risk factors and trends of obstructive coronary artery disease (CAD) in Indian females <45 years of age compared to males of the same age group who underwent percutaneous coronary intervention (PCI).

**Materials and Methods:**

This was a retrospective, observational, multi-centre study of young Indian females and males (<45 years) who underwent PCI as per the guidelines at three high-volume centres in India.

**Results:**

In a group of 3656 patients under the age of 45 who had PCI, 3.1% of those with obstructive CAD were young women (*n* = 113), while 6.9% were young men (*n* = 254). Traditional risk factors such as hypertension (p = 0.73), diabetes (p = 0.61), and family history of premature CAD (p = 0.63) were equally common in both genders, whereas dyslipidaemia (p < 0.001), overweight (p < 0.006), smoking (p = 0.004) and, alcoholism (p < 0.001) were more common in young males. Acute coronary syndrome was the most common clinical presentation. Single-vessel disease was common, with the involvement of the left anterior descending artery as the most common angiographic feature. The prevalence of cardiogenic shock was 4.4% in females and 4.1% in males, while the in-hospital mortality rate was 1.77% in young females and 2% in young males.

**Conclusions:**

Obstructive CAD in young men and women accounted for 10% of all CAD cases requiring PCI. Although men account for the majority of patients, CAD in women under the age of 45 is not uncommon. Traditional risk factors are becoming more prevalent in younger women.

## Graphical abstract

**Figure:**
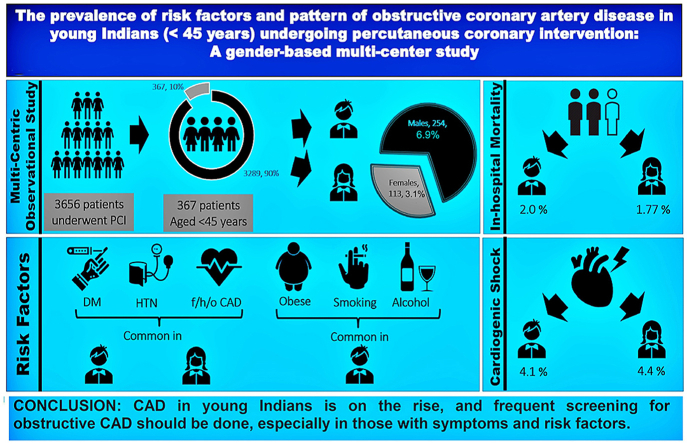

## 1 Introduction

In developing countries such as India, cardiovascular diseases (CVD) have surpassed cancer as the leading cause of death and morbidity.^1^ In reference to the United Nations Declaration on Noncommunicable Diseases (NCDs) in 2011, the World Health Organization (WHO) defined a target of lowering the likelihood of premature death from NCDs, such as CVDs, by 25% by 2025.^2^ It is estimated that 55 million people died in the world in 2017, with 17.7 million as a result of CVD, with the number expected to double by 2030.^3^ However, premature obstructive CAD has become a prime concern for patients, practitioners, politicians, and policymakers in India. Coronary artery disease (CAD) in young people refers to CAD that occurs before the age of 45. Various research, meanwhile, has suggested an age restriction ranging from 35 to 55 years in the spectra of CAD in young people.^4^ With changing lifestyles, the onset of traditional risk factors at the early stages of life is the major culprit for developing CAD at an early age. For several years, the significance of CAD in young women was underestimated. There are some sex-related variations in CAD, and symptoms of CAD in women, regardless of age, have low specificity even in the absence of risk factors.^5^ Moreover, the presence of obstructive CAD in young women is usually neglected, due to the notion that women are at a very low risk of developing CAD at a young age. Furthermore, even though women with obstructive CAD have a less extensive disease than men, the former have more severe symptoms regardless of the severity of the disease.^6^ Furthermore, when comparing young women to equally aged men, gender disparities in post-acute coronary syndrome mortality risk are even more pronounced. Furthermore, following reperfusion procedures, women have a substantially higher incidence of adverse effects such as bleeding complications.^7^

So, in a multicenter retrospective analysis, we aimed to see how common risk factors, the clinical profile and patterns of obstructive CAD, and in-hospital outcomes differed between women under 45 years old who had percutaneous coronary intervention (PCI) compared to their male counterparts.

## 2 Methodology

This was a retrospective, observational, multi-centre study of 3656 patients who had obstructive coronary artery disease and were admitted to the intensive coronary care unit and underwent PCI according to guidelines for established obstructive CAD from July 2016 to December 2018, at three high-volume corporate hospitals from three states (Telangana, Maharashtra, and Gujarat) of India. Ethical clearance from the institutional ethical committees was obtained.

## 3 Collection of data

The digital medical record system of the hospital was the source of all data which was collected either by assigned personnel or by qualified research coordinators. Information collected through the proforma included history, including family history and details of risk factors. Present and past medical records were used to collect information about the patient's clinical presentation, including diagnostic tests such as electrocardiogram and echocardiography, treatments, and laboratory tests such as blood sugar levels, and fasting lipid profiles (after 12 h of fasting), thyroid profile, and collagen vascular profile. The obstetric history was obtained from all the females of the childbearing age group, including the history of usage of oral contraception. For patients with obstructive CAD who underwent PCI, detailed information on demographic variables, conventional coronary risk factors such as hypertension, diabetes mellitus, dyslipidemia, family history of premature cardiovascular disease, obesity, smoking, and alcohol use is defined below.

1. **Hypertension** was identified as a patient receiving care for high blood pressure or having a systolic pressure of 140 mmHg and/or a diastolic pressure of 90 mmHg.
2. **Diabetes** was described as fasting blood glucose equal to or greater than 126 mg/dl, 2-h postprandial blood glucose greater than 200 mg/dl, HbA1c 6.5%, and/or patients with a history of diabetes mellitus or who are seeking anti-diabetic therapy.
3. **Dyslipidemia** was described as patients on lipid-lowering therapy or with plasma lipids in the following ranges: Total cholesterol greater than 240 mg/dl, triglycerides greater than 150 mg/dl, low-density lipoprotein (LDL) cholesterol greater than 130 mg/dl, and high-density lipoprotein (HDL) cholesterol lesser than 40 mg/dl.
4. A **family history of premature CAD** was defined as having a first-degree relative who was diagnosed with CAD before the age of 55 in a male relative or 65 in a female relative.
5. Those with a body mass index (BMI) above 23 kg/m^2^ were classified as **obese.** Broca's formula was used to measure the BMI of all of the patients.
6. A **smoker** was described as anyone who smoked one or more cigarettes per day on average for at least a year. Ex-smokers were patients who had not smoked for more than 12 months. The study did not look into the history of passive smoking.
7. **Chronic alcoholism** is defined as a chronic disorder influenced by genetic, psychosocial, and environmental causes, marked by a prolonged period of regular, excessive alcohol consumption as well as reluctance to regulate drinking once it has started.

The information related to other medical comorbidities (non-traditional risk factors) such as hypothyroidism, Takayasu arteritis, collagen vascular diseases, rheumatic heart disease, and spontaneous coronary artery dissection was collected as per the definitions below:

1. **Hypothyroidism** is defined as a thyroid hormone deficiency with elevated TSH and a reduced T4 or free thyroxine index.
2. **Takayasu arteritis** was classified using the American College of Rheumatology classification, which required the presence of three of the six criteria.^8^
3. **Collagen vascular diseases (also known as Connective tissue diseases)** are caused by an auto-immune response to collagen and there may be a vascular component. There are a diverse group of disorders characterised by the presence of autoantibodies, including systemic sclerosis, rheumatoid arthritis, systemic lupus erythematosus, dermatomyositis/polymyositis, ankylosing spondylitis, Sjögren syndrome, undifferentiated connective tissue disease, and mixed connective tissue disease.
4. Following the confirmation of a history of rheumatic fever or typical echocardiographic features, a diagnosis of **rheumatic heart disease** is made based on the presence of permanent heart valve damage.
5. **Spontaneous coronary artery dissection** is defined as a non-atherosclerotic spontaneous coronary artery dissection caused by a tear in the tunica intima of the blood vessel, with blood entering and separating the layers of the arterial wall and a pathognomonic angiographic feature of the identifiable false lumen with a linear filling defect or dissection flap ranging from contrast stasis in the false lumen to stenosis to complete occlusion.

## 4 Inclusion criteria

The angiographic information like the number of vessels involved, the severity of lesions, and details about the PCI were all gathered in detail. The patients were classified as having single-vessel disease, double-vessel disease, or triple-vessel disease, determined by the number of major epicardial coronary arteries affected (left anterior descending artery [LAD], left circumflex artery [LCX], and right coronary artery [RCA]). Obstructive CAD was identified as the presence of at least one major epicardial coronary artery or its branch with a lumen diameter narrowing of more than 70% and stenosis of more than 50% of the left main coronary artery (LMCA).^9^ Of all patients, the clinical-angiographic information of young females and males of 45 years of age was included in the analysis.

## 5 Statistical analysis

All data was represented as counts and percentages. Descriptive statistics were used to interpret the results, including means, medians, ranges, standard deviations, and percentages. To compare differences between the categories, the Chi-square test, the student *t*-test, and the odds ratio calculator were used. All significance tests were two-tailed, and statistical significance was described as a *P* value of less than 0.049. The Windows SPSS 16.0 software (SPSS, Chicago, IL, USA) was used to conduct all statistical analyses.

## 6 Results

### 6.1 Study population

A total of 3656 PCIs (females: 37.6%; males: 62.4%) were conducted during the study period, with young patients (age less than 45 years) with obstructive CAD accounting for 367 (10%) of those who underwent PCIs. The 367 patients included 254 (69.2%) males and 113 (30.8%) females. Young females accounted for 8.2% of all females (113 out of 1374) studied, whereas young males represented 11.1% of males studied (254 out of 2282). The prevalence of obstructive CAD necessitating PCI was found to be 3.1% in young females and 6.9% in young males. Male patients had a mean age of 37.4 (± 7.3) years, while female patients had a mean age of 41.1 (± 3.8) years. Male patients were more overweight, with a baseline BMI of 27.4 (± 4.27) kg/m2 compared to female patients at 24.7 (± 3.91) kg/m2.

### 6.2 Prevalence of risk factors in young with obstructive CAD

**Fig. 1:**
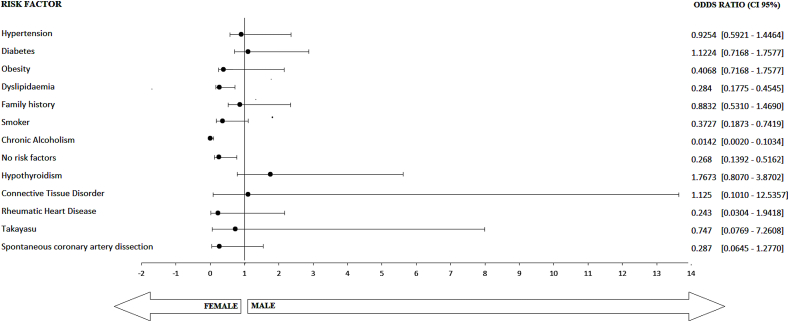
Forest plot analysis of the distribution of various risk factors and associated medical conditions among young Indians presented with obstructive CAD those who underwent PCI. Abbreviations: CAD = Coronary artery disease; PCI = Percutaneous coronary intervention; CI = Confidence Interval.

Over 40% of women and men had hypertension (p = 0.733) and diabetes (p = 0.613), but the differences were statistically insignificant. Overweight (p = 0.006), dyslipidemia (p < 0.001), smoking (p = 0.004), and chronic alcoholism (p < 0.001) were less frequent amongst women compared to men. Importantly, fewer women than men had no risk factors (10.6% vs. 30.7%, p = 0.003). Various risk factors such as hypertension, diabetes, and dyslipidemia were under control in 45.7% of young patients using targeted therapies in this study. Two risk factors were present in 16.1% of young females and 33.4% of young males, whereas more than three risk factors were present in 3% of young females and 10% of young males. 10.4 percent of females reported using oral contraceptive pills in the past. Hypothyroidism (p = 0.15), spontaneous coronary artery dissection (p = 0.08), connective tissue disorders (p = 0.92), rheumatic valvular heart diseases (p = 0.15), and Takayasu arteritis (p = 0.80) were the isolated occurrences of predisposing medical conditions (Non-traditional risk factors for CAD). Overall, 15.04% of women (17 of 113) and 17.7% of males (45 of 254) were diagnosed with one or more of these associated medical conditions. However, this difference was not statistically significant (p = 0.675). Table 1 and Fig. 1 shows how often different risk factors for CAD were found in the sample population.

**Table 1:** Baseline demographics and distribution of risk factors in patients included in the study.

Total no. of PCI during the study period, n (%)	3656						
Risk factors		Females (*n* = 113)	Males (*n* = 254)	Total	*p*-value	Odds ratio	95% confidence interval
Hypertension, n(%)	Yes	49 (43.3%)	115 (45.3%)	164	0.73	0.92	0.59–1.44
	No	64	139	203			
Diabetes, n(%)	Yes	49 (43.4%)	103 (40.5%)	152	0.61	1.12	0.71–1.75
	No	64	151	215			
Overweight, n(%)	Yes	23 (20.4%)	98 (38.6%)	121	<0.006	0.40	0.71–1.75
	No	90	156	246			
Dyslipidemia, n(%)	Yes	36 (31.9%)	158 (62.2%)	194	<0.001	0.28	0.17–0.45
	No	77	96	173			
Family history of premature CAD, n(%)	Yes	28 (24.8%)	69 (27.1%)	97	0.632	0.88	0.53–1.47
	No	85	185	270			
Smoking, n(%)	Yes	11 (9.7%)	57 (22.4%)	68	0.004	0.37	0.18–0.74
	No	102	197	299			
Chronic Alcoholism, n(%)	Yes	1 (0.9%)	98 (38.6%)	99	<0.001	0.01	0.002–0.10
	No	112	156	268			
No risk factors, n(%)	Yes	12 (10.6%)	78 (30.7%)	90	0.003	0.26	0.14–0.51
	No	101	176	277			
**Associated Medical Conditions**							
Hypothyroidism, n(%)	Yes	12 (10.6%)	16 (6.2%)	28	0.15	1.76	0.80–3.87
	No	101	238	339			
Connective Tissue Disorder, n(%)	Yes	1 (0.9%)	2 (0.78%)	3	0.92	1.12	0.10–12.53
	No	112	252	364			
Rheumatic Heart Disease, n(%)	Yes	1 (0.9%)	9 (3.5%)	10	0.15	0.24	0.03–1.94
	No	112	245	357			
Takayasu Arteritis, n(%)	Yes	1 (0.9%)	3 (1.2%)	4	0.80	0.74	0.07–7.26
	No	112	251	363			
Spontaneous coronary artery dissection, n(%)	Yes	2 (1.8%)	15 (5.9%)	17	0.08	0.29	0.06–1.27
	No	111	239	350

### 6.3 The pattern of obstructive CAD

**Fig. 2:**
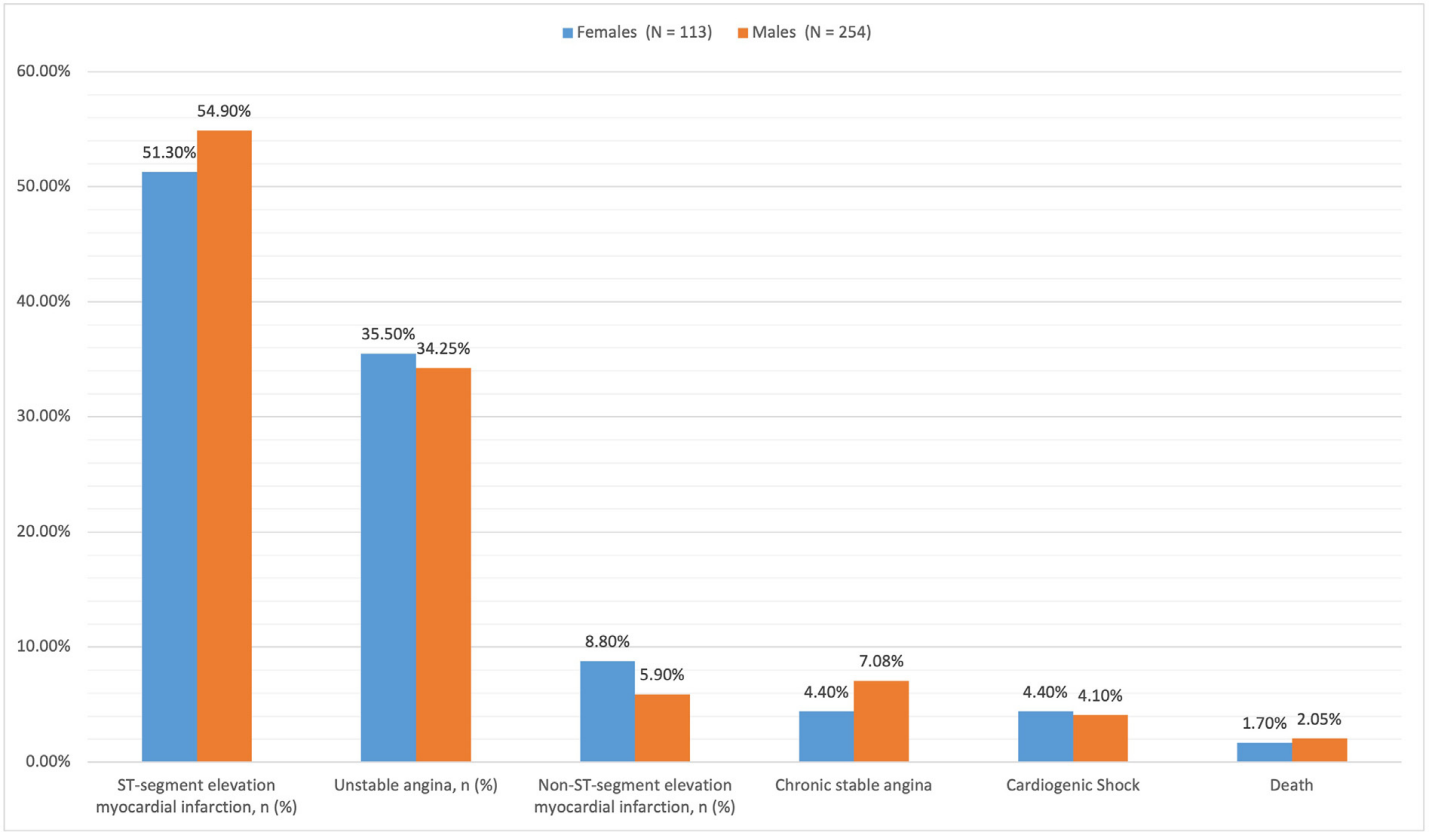
Clinical presentation of coronary artery disease in young Indians. Abbreviations: STEMI = ST-elevation myocardial infarction; NSTEMI = Non-ST elevation myocardial infarction.

Acute coronary syndrome was the most common clinical presentation in both categories (95.6 in young females vs. 95% in young males; average 95.3%), with ST-segment elevation myocardial infarction (STEMI) being the most common, followed by unstable angina and, least often, non-ST-segment elevation myocardial infarction (NSTEMI). Chronic coronary syndrome in the form of chronic stable angina was a less common presentation in young Indians.There was no statistically significant relationship between clinical manifestations, mortality, and gender observed. The details of the clinical presentation and angiographic profile of the sample population are demonstrated in Table 2 and Fig. 2. The pattern of epicardial vessel involvement was similar in both men and women, with most subjects having single-vessel disease. Triple vessel disease was not seen in women and occurred in 5.5% of men. Among the cases classified as single-vessel disease, the LAD was the most commonly involved, followed by the RCA and then the LCX. An average of 2.7% of young people had isolated LMCA disease as a single-vessel condition. The diagonal artery was the coronary vessel that was least affected. The most common double-vessel disease was LAD and RCA involvement, followed by LAD and LCX involvement. The angiographic patterns in related comorbid disorders such as Takayasu arteritis, collagen vascular disorders, and chronic rheumatic heart disease were not different from those in atherosclerotic CAD. Also, there was no statistical gender-based difference in patients who had spontaneous coronary artery dissections. The females who had SCAD were oral contraceptive users. During hospitalization, cardiogenic shock occurred in 4.4% of young females and 4.1% of young males. The mortality rate was 1.7% in young females while 2.05% of males died secondary to CAD. All the deaths were related to STEMI and cardiogenic shock as their clinical presentation.

**Table 2:** Clinical and diagnostic presentation of patients included in the study.

Variables	Females (N = 113) (8.2%)	Males (N = 254) (11.1%)	*p*-value
**Clinical presentation**			
ST-segment elevation myocardial infarction, n (%)	58 (51.3%)	134 (54.9%)	0.580.82
Unstable angina, n (%)	40 (35.5%)	87 (34.25%)	
Non-ST-segment elevation myocardial infarction, n (%)	10 (8.8%)	15 (5.9%)	
Acute coronary syndrome, n(%)	108 (95.6%)	236 (95.05%)	
Chronic stable angina, n (%)	05 (4.4%)	18 (7.08%)	
Cardiogenic Shock	5 (4.4%)	15 (4.1%)	
Death	2 (1.7%)	5 (2.05%)	
**No. of diseased vessels**			
Single vessel disease (SVD), n (%)	84 (74.3%)	162 (63.7%)	0.19
Double vessel disease (DVD), n (%)	29 (25.7%)	78 (30.7%)	
Triple vessel disease (TVD), n(%)	0	14 (5.5%)	
**Distribution of SVD**			
Left anterior descending artery, n (%)	38 (45.2%)	78 (48.1%)	0.88
Right coronary artery, n (%)	21 (25.0%)	44 (27.2%)	
Left circumflex artery, n (%)	19 (22.6%)	28 (17.3%)	
Diagonal artery, n (%)	03 (3.6%)	05 (3.1%)	
Left main coronary artery, n (%)	03 (3.6%)	07 (4.3%)	
**Distribution of DVD**			
LAD/RCA, n (%)	17 (58.6%)	28 (35.9%)	0.30
LAD/LCX, n (%)	05 (17.3%)	15 (19.2%)	
Ramus/RCA, n (%)	02 (6.9%)	04 (5.1%)	
LAD/Diagonal, n (%)	02 (6.9%)	14 (17.9%)	
LCX/RCA, n (%)	02 (6.9%)	10 (12.8%)	
Diagonal/LCX, n (%)	01 (3.4%)	07 (9.0%)

## 7 Discussion

### 7.1 Correlation between risk factors and premature CAD

It is a well-known fact that the higher incidence of proven risk factors is to blame for the CAD epidemic in India. There is little published data from recent Indian studies on the determinants of obstructive CAD in young Indians. In a study conducted in south India by Iragavarapu et al, the average age of CAD in young people was 36.11 years, and males exceeded females, comparable to our findings.^10^ Earlier studies stated that women develop CAD at an age 10 years later than men.^11^ Traditional risk factors such as hypertension, diabetes, and dyslipidemia, which were once more prevalent in males, are now on the rise in young Indian women, as evidenced by recent Indian population studies, and our findings in the current study are also comparable to young males.^12^^,^^13^ Also, the INTERHEART study reported that hypertension and diabetes were more imminent risk factors in young Indian women than in men.^14^ In our sample, there were no significant differences in the risk factor profiles of patients from three states. In young Indians, the prevalence of risk factors such as hypertension, diabetes, and abnormalities in lipid parameters such as low high-density lipoprotein, high total cholesterol, triglycerides, low-density lipoprotein, and lipoprotein (a) is usually linked to the development of premature obstructive CAD.^15^ Even though hormone protection could delay atherosclerosis in women, our study found that at a young age, the many risk factors completely cancel out the benefit.

### 7.2 Characteristics of CAD in young

Men and women present differently towards obstructive CAD in terms of risk factors, genetics, socio-cultural processes, biological factors, and pathophysiology. According to several irrefutable pieces of evidence, women with presumed ischemia are often undervalued. Young women have about a 50% higher risk of mortality than young men, probably because diagnostic and therapeutic management is minimal in women at a young age.^16^^,^^17^ Because of resting ST-T segment variations, lower electrocardiogram voltages, and some hormonal variables, the treadmill test has very poor sensitivity in women.^18^ Because of the higher prevalence of atypical symptoms, the restricted usefulness of non-invasive diagnostic testing, and the regular identification of normal/nonobstructive CAD on angiography, identifying the predictors of obstructive CAD in younger females before invasive testing is critical.

The prevalence of obstructive CAD in young women aged 45 years was found to be 3.1% in our study, which is slightly lower, possibly due to the inclusion of patients of a younger age group, than the 4.4% incidence reported by Bhatt et al in women aged 50 years.^6^ However, in our study, females with risk factors (89.4%) had a higher prevalence of obstructive CAD than those without risk factors (10.6%), and so the presence of risk factors in young females is one of the strongest predictors of CAD.

On computed tomography of the coronary arteries, it has been observed that women have smaller coronary artery diameters than men.^19^ Han et al studied men and women with early CAD and observed that men had excessive atheroma and epicardial endothelial dysfunction, whereas women had more microvascular dysfunction.^20^ In an autopsy study conducted by Vaideeswar et al, 88.4% of young patients had obstructive CAD caused by atherosclerosis, with females accounting for 15.5%. Predictably, the LAD was predominantly involved, as was the appearance of fibrous plaques. Thrombosis caused by plaque erosion was the most frequent cause, seen in 36.9% of patients regardless of plaque morphology in both genders. Acute coronary syndrome was found in 61.9% of the patients.^21^

Acute coronary syndrome was the commonest presentation in the young in our study, like in the CREATE registry, which recorded STEMI in 61% of patients. STEMI was present in an average of 52.3% of men and women in our study.^18^ Huang et al observed that obstructive CAD of the LMCA and LAD is more common in young males. However, in our analysis, the pattern of single-vessel disease was comparable in both genders.^22^ Iragavarapu et al found that single-vessel disease was the most prevalent form of vascular disease in their 120 young South Indian patients who presented with the acute coronary syndrome (10.4%) at a tertiary care hospital, analogous to our findings.^6^ Our analysis found about 5.5% of TVD, which is consistent with reported data from Jamil et al^23^ and Iragavarapu et al^10^

### 7.3 Prevention of CAD in young Indians

The presence of comorbidities such as hypertension, diabetes, dyslipidemia, and obesity, can be controlled and corrected using preventive measures. Females are less likely to smoke, which is a trait that we can eradicate. Younger age groups have a wider window of opportunity for the prevention of premature CAD. When it comes to analysing risk factors in women, it was observed that the prevalence of risk factors was more prominent in post-menopausal women than in premenopausal women.^24^ As a result, pre-menopausal women have a greater benefit when it comes to taking preventive steps against these risk factors. Targeted regulation of hypertension, obesity, lipid levels, and glycemia is needed, with a focus on counselling the young on the mitigation of these risk factors to reduce the burden of disease. It, therefore, indicates that young Indians have an additional ten years to implement preventive measures to successfully fight the menace of CAD. This can be done by creating awareness among young individuals about modifiable risk factors. Awareness programmes and campaigns can be organized, and seminars and webinars can be conducted by specialised physicians so that the general population gets knowledge about the prevention of obstructive CAD from a young age, because “prevention is better than cure”.^25^^,^^26^

### 7.4 Study limitations

Our research had a few limitations: 1) Our study was a brief retrospective analysis of data from young Indian patients hospitalised for PCI with significant underlying CAD. It is not an epidemiological review for determining CAD prevalence in society. Our findings are a reflection of India's three states and not the whole country. 2) Also, we did not study time to presentation, SYNTAX score and, details of the PCIs, such as the number of stents used, procedural complications, stent thrombosis, etc. 3) We haven't looked at psychosocial influences, physical activity, sedentary behaviour patterns, nutritional awareness, thyroid status, or the effect of oral contraceptives in this young age group. 4) Finally, our study had no follow-up data as the study aimed to determine the prevalence of risk factors and obstructive coronary disease in young Indian women. Finally, some of the variables, such as the family history of premature CAD, may be skewed because we were unable to conduct genetic studies.

### 7.5 What is already known?

CAD is becoming more common in young Indians, particularly in young females. In the modern world, the onset of traditional risk factors at an early age is the primary cause of the development of CAD at an early age. For a long time, the significance of coronary artery disease (CAD) among young women was undervalued. Even in the absence of risk factors, the symptoms of CAD in women have low specificity, and there are some sex-related variations in CAD.

### 7.6 What this study adds?

• Though men make up the majority of patients in this age group, CAD in females under the age of 45 is not uncommon.
• Approximately 9–11% of all CAD cases are caused by obstructive CAD in young people, which is a distinct entity with a distinctive risk factor profile, clinical appearance, angiographic profile, and therapeutic outcome.
• Traditional risk factors including hypertension, diabetes, abdominal obesity, smoking, and alcohol intake are on the rise in younger women and are on par with men.
• STEMI is a typical presentation of CAD, and single-vessel disease is a more common form of angiographic pattern in the young, and the most common culprit vessel is the LAD.
• Younger patients are treated with PCI as the primary modality, and they perform better with few complications.
• Although coronary artery disease is less common in young adults, it still presents a significant challenge for both the patient and the attending specialist.
• More attention should be paid to routinely screening and counselling the young on the regulation of these modifiable risk factors in order to reduce the incidence of CAD and the mortality associated with it.

## Funding

There is no source of funding for this article.

## Credit author statement

PJ and KJ are Consultant Cardiologist who treated patients, and responsible for the writing manuscript, collection, and preparation of the article. AP assisted in the review of the manuscript, statistical calculations, and grammatical and spelling corrections. RP and KDC shared their cases and reviewed manuscript independently.

## Declaration of competing interests

As an author, I declare that there is no financial or non-financial conflict/competing of interests. This manuscript is not submitted to any journal before for publication as a part or complete version. I give complete consent and rights to the journal for its publication. Informed consent was obtained from a participant included in the study. All procedures performed in studies involving human participants were in accordance with the ethical standards of the institutional and/or national research committee and with the 1964 Helsinki declaration and its later amendments or comparable ethical standards.

## References

[1] Gupta R., Guptha S., Sharma K.K., Gupta A., Deedwania P. (2012). Regional variations in cardiovascular risk factors in India: India heart watch. World J Cardiol.

[2] Prabhakaran D., Singh K., Roth G.A., Banerjee A., Pagidipati N.J., Huffman M.D. (2018).

[3] Yusuf S., Joseph P., Rangarajan S. (2020). Modifiable risk factors, cardiovascular disease, and mortality in 155 722 individuals from 21 high-income, middle-income, and low-income countries (PURE): a prospective cohort study. Lancet.

[4] Aggarwal A., Srivastava S., Velmurugan M. (2016). Newer perspectives of coronary artery disease in young. World J Cardiol.

[5] Vogel B., Acevedo M., Appelman Y. (2021). The Lancet women and cardiovascular disease Commission: reducing the global burden by 2030. Lancet.

[6] Bhatt P., Parikh P., Patel A. (2015). Unique aspects of coronary artery disease in Indian women. Cardiovasc Drugs Ther.

[7] Claassen M., Sybrandy K.C., Appelman Y.E., Asselbergs F.W. (2012). Gender gap in acute coronary heart disease: myth or reality?. World J Cardiol.

[8] Arend W.P., Michel B.A., Bloch D.A. (1990). The American College of Rheumatology 1990 criteria for the classification of Takayasu arteritis. Arthr Rheum.

[9] Members W.C., Levine G.N., Bates E.R. (2011). ACCF/AHA/SCAI guideline for percutaneous coronary intervention. Circulation.

[10] Iragavarapu T., Radhakrishna T., Babu Kj, Sanghamitra R. (2019). Acute coronary syndrome in young - a tertiary care centre experience with reference to a coronary angiogram. J Pract Cardiovasc Sci.

[11] Kornowski R, Lansky AJ, Mintz GS, et al. Comparison of Men versus Women in Cross-Sectional Area Luminal Narrowing, the Quantity of Plaque, Presence of Calcium in Plaque, and Lumen Location in Coronary Arteries by Intravascular. 10.1016/s0002-9149(97)00206-3 9202348

[12] Sharma A., Dar M., Iqbal M., Tramboo N. (2020). Gender-based differences in coronary artery disease: a prospective observational study from a North Indian state. Heart India.

[13] Mahajan K., Kandoria A., Bhardwaj R., Kandoria M. (2019). Obstructive coronary artery disease in women presenting with ischemic chest pain: prevalence and risk determinants. J Pract Cardiovasc Sci.

[14] Yusuf S., Hawken S., Ôunpuu S. (2004). Effect of potentially modifiable risk factors associated with myocardial infarction in 52 countries (the INTERHEART study): case-control study. Lancet.

[15] Pathak L.A., Shirodkar S., Ruparelia R., Rajebahadur J. (2017). Coronary artery disease in women. Indian Heart J.

[16] Vaccarino V., Krumholz H.M., Berkman L.F., Horwitz R.I. (1995). Sex differences in mortality after myocardial infarction: is there evidence for an increased risk for women?. Circulation.

[17] Stangl V., Baumann G., Stangl K. (2002). Coronary atherogenic risk factors in women. Eur Heart J.

[18] Mosca L., Barrett-Connor E., Wenger N.K. (2011). Sex/gender differences in cardiovascular disease prevention: what a difference a decade makes. Circulation.

[19] Dalal J., Hiremath M.S., Das M.K., Desai D.M., Chopra V.K., Biswas A.D. (2016). Vascular disease in young Indians (20-40 years): role of ischemic heart disease. J Clin Diagn Res.

[20] Han S.H., Bae J.H., Holmes D.R. (2008). Sex differences in atheroma burden and endothelial function in patients with early coronary atherosclerosis. Eur Heart J.

[21] Vaideeswar P., Tyagi S., Singaravel S. (2019). Pathology of atherosclerotic coronary artery disease in the young Indian population. Forens Sci Res.

[22] Huang J, Qiao S, Xu B, et al. Clinical Characteristics and Outcome Comparison between Young (< Or= 45 Years) Female and Male Patients with Coronary Artery Disease Undergoing Percutaneous Coronary Intervention. [Zhonghua xin xue guan bi]. 20450568

[23] Jamil G., Jamil M., Alkhazraji H. (2013). Risk factor assessment of young patients with acute myocardial infarction. Am J Cardiovasc Dis.

[24] Zhou H., Zhang C., Ni J., Han X. (2019). Prevalence of cardiovascular risk factors in non-menopausal and postmenopausal inpatients with type 2 diabetes mellitus in China. BMC Endocr Disord.

[25] Iyengar S., Gupta R., Ravi S. (2017). Premature coronary artery disease in India: coronary artery disease in the young (CADY) registry. Indian Heart J.

[26] Gupta R., Puri V., Narayan V., Saran P., Dwivedi S., Singh S. (1999). Cardiovascular risk profile in Indian women. Indian Heart J.

